# Genomic characterization and outcome evaluation of kinome fusions in lung cancer revealed novel druggable fusions

**DOI:** 10.1038/s41698-021-00221-z

**Published:** 2021-09-10

**Authors:** Binghao Li, Hao Qu, Jing Zhang, Weibo Pan, Meng Liu, Xiaobo Yan, Xin Huang, Xuexin He, Dong Lin, Sisi Liu, Ruting Guan, Yong Wu, Qiuxiang Ou, Hua Bao, Youbin Xu, Xue Wu, Yang Shao, Nong Lin

**Affiliations:** 1 Bone Metastasis Service, Department of Orthopaedics, The Second Affiliated Hospital of Zhejiang University School of Medicine, Hangzhou, Zhejiang China; 2 Institute of Orthopaedic Research, Zhejiang University, Hangzhou, Zhejiang China; 3 Department of Thoracic Oncology, Fujian Cancer Hospital, Fujian Medical University Cancer Hospital, Fuzhou, Fujian China; 4 Department of Medical Oncology, The Second Affiliated Hospital of Zhejiang University School of Medicine, Hangzhou, Zhejiang China; 5 Nanjing Geneseeq Technology Inc., Nanjing, Jiangsu China; 6 Department of Medical Oncology, Zhangzhou Zhengxing Hospital, Zhangzhou, Fujian China

**Keywords:** Cancer, Molecular biology

## Abstract

Kinase fusions represent an important type of somatic alterations that promote oncogenesis and serve as diagnostic markers in lung cancer. We aimed to identify the landscape of clinically relevant kinase fusions in Chinese lung cancer and to explore rare kinase rearrangements; thus, providing valuable evidence for therapeutic decision making. We performed genomic profiling of 425 cancer-relevant genes from tumor/plasma biopsies from a total of 17,442 Chinese lung cancer patients using next generation sequencing (NGS). Patients’ clinical characteristics and treatment histories were retrospectively studied. A total of 1162 patients (6.66%; 1162/17,442) were identified as having kinase fusions, including 906 adenocarcinomas (ADCs) and 35 squamous cell carcinomas (SCCs). In ADC, 170 unique gene fusion pairs were observed, including rare kinase fusions, *SLC12A2-ROS1*, *NCOA4-RET*, and *ANK3-RET*. As for SCC, 15 unique gene fusions were identified, among which the most frequent were *EML4-ALK* and *FGFR3-TACC3*. Analyses of oncogenic mutations revealed a dual role for the gene fusions, *CCDC6-RET* and *FGFR3-TACC3*, in driving oncogenesis or serving as acquired resistance mechanisms to kinase inhibitors. In addition, our real-world evidence showed that patients with recurrent kinase fusions with low frequency (two occurrences) could benefit from treatment with kinase inhibitors’ off-label use. Notably, patients with stage IV ADC who had novel *RORB-ALK* or *AFF2-RET* fusions, but no other known oncogenic driver mutations, demonstrated favorable clinical outcomes on tyrosine kinase inhibitors. Our data provide a comprehensive overview of the landscape of oncogenic kinase fusions in lung cancer, which assist in recognizing potentially druggable fusions that can be translated into therapeutic applications.

## Introduction

Lung cancer is the leading cause of cancer-related death worldwide, with a 5-year survival rate of less than 21%^1^. There are two major histological groups of lung cancer, including small cell lung cancer (SCLC) and non-SCLC (NSCLC). NSCLC accounts for approximately 85% of all lung cancers^1^ and can be further divided into different subtypes, among which, the most common is adenocarcinoma (ADC), followed by squamous cell carcinoma (SCC), adeno-squamous cell carcinoma (ASC), and large cell carcinoma (LCC). However, the molecular features of these subtypes are significantly distinct. For that reason, the identification of oncogenic driver genes and novel therapeutic targets is highly important for lung cancer treatment.

Kinases activated by gene fusions have been reported to be major classes of oncogenic drivers in lung cancer, which are produced by translocation or structural chromosome rearrangements, and function as potential targets of anticancer drugs^2,3^. Advances in next-generation sequencing (NGS) technologies have enabled the characterization of kinase fusions among different lung cancer subtypes, the identification of concurrent cancer-relevant alterations, and the identification of novel driver fusions.

In this study, we comprehensively analyzed DNA-seq data from 17,442 Chinese lung cancer patients, described the clinical and pathological characteristics of patients with different kinase fusions, and distinguished kinase fusion types between ADC and SCC. Additionally, we deeply studied the fusion breakpoint preferences of kinases and analyzed treatment-relevant mutations that co-occurred with primary recurrent kinase fusions. Furthermore, we identified multiple novel druggable fusions, which benefited from tyrosine kinase inhibitor (TKI) treatment. These studies facilitated a deep understanding of the unique clinical and molecular features, and the outcomes of lung cancer patients with kinase fusions.

## Results

### The landscape of kinase fusions in lung cancer

**Fig. 1:**
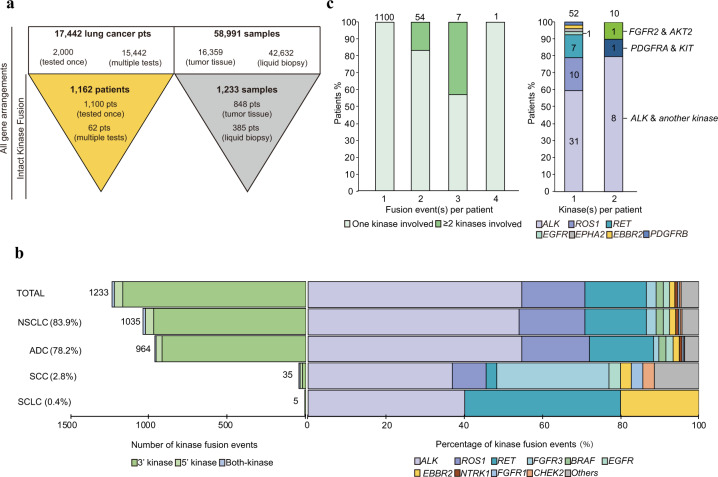
Overview of kinase fusions in lung cancer patients. **a** Pipeline for kinase fusion calling and filtering. **b** The left bar plot shows the distribution of the three kinase fusion categories in different lung cancer subtypes. The number of events in each condition is indicated to the left of each bar. The right bar plot shows the percentage of the kinase genes involved across different lung cancer subtypes. **c** The left bar graph shows the number of kinase fusions in one patient. The right bar graph shows the kinase genes of patients who carried more than one kinase fusion. The number of patients in each condition is indicated on the top of each bar.

DNA-seq data from 17,442 lung cancer patients were analyzed, among which 2000 were unique cases while 15,442 patients had been tested more than once (Fig. 1a). After a series of filters and identifications, 1233 fusions with intact kinase domains were observed in 1162 patients with various histological subtypes, including ADC (78%; 906/1162), SCC (3.0%; 35/1162), adeno-squamous carcinoma (ASC; 0.8%; 9/1162), LCC (0.3%; 4/1162), and SCLC (0.4%; 5/1162) (Table 1). Sixty-one patients had bone metastasis. The median age of patients with ADC was 53 years (range: 21–95 years), which was significantly younger than that of other NSCLC subtypes (*p* < 0.001). In addition, the prevalence of males was higher than females (65.7% vs. 34.3%, respectively) in SCC patients.

**Table 1:** Summary of the demographic and clinical characteristics of the kinase fusion cohort in this study.

	Total	NSCLC					SCLC	Unknown
		ADC	SCC	ASC	LCC	Unknown		
Cases	17,442	10,932	1353	162	63	217	549	4166
Cases with kinase fusions	1162	906	35	9	4	22	5	181
Median age (range), years	53 (21–95)	53 (21–95)	60 (31–82)	60 (37–70)	61 (44–64)	60 (39–82)	59 (46–64)	54 (26–86)
<60, *n* (%)	713 (61.4)	585 (64.6)	16 (45.7)	4 (44.4)	1 (25.0)	10 (45.4)	3 (60.0)	94 (51.9)
≥60 years, *n* (%)	366 (31.5)	277 (30.6)	17 (48.6)	5 (55.6)	2 (50.0)	11 (50.0)	2 (40.0)	52 (28.7)
Unknown	83 (7.1)	44 (4.8)	2 (5.7)	0	1 (25.0)	1 (4.6)	0	35 (19.4)
Sex, *n* (%)								
Male	602 (51.8)	434 (47.9)	23 (65.7)	4 (44.4)	1 (25.0)	14 (63.6)	3 (60.0)	81 (44.7)
Female	560 (48.2)	472 (52.1)	12 (34.3)	5 (55.6)	3 (75.0)	8 (36.4)	2 (40.0)	100 (55.3)
Stage, *n* (%)								
I	21 (1.8)	19 (2.1)	0	1 (11.1)	0	0	0	1 (0.5)
II	20 (1.7)	18 (2.0)	2 (5.7)	0	0	0	0	0
III	68 (5.9)	60 (6.6)	2 (5.7)	0	0	1 (4.6)	0	5 (2.8)
IV	535 (46.0)	457 (50.4)	14 (40.0)	5 (55.6)	2 (50.0)	10 (45.4)	3 (60.0)	44 (24.3)
Unknown	518 (44.6)	352 (38.9)	17 (48.6)	3 (33.3)	2 (50.0)	11 (50.0)	2 (40.0)	131 (72.4)

Abbreviations: *NSCLC* non-small-cell lung cancer, *SCLC* small cell lung cancer, *ADC* adenocarcinoma, *SCC* squamous cell carcinoma, *ASC* adeno-squamous carcinoma, LCC large cell carcinoma.

We classified the 1233 kinase fusion events into three categories, including 1165 with a kinase at the 3′ end (3′ kinase), 57 with a kinase at the 5′ end (5′ kinase) and 11 fusions in which both partners were kinases (both-kinase) (Fig. 1b). In both-kinase patients, *TRIM24-BRAF* fusions were the most frequent (54.4%; 6/11; data not shown) and reported to be sensitive to a MEK inhibitor^4^. *ALK* (54.8%; 674/1233), *ROS1* (16.2%; 200/1233), *RET* (15.8%; 195/1233), and *FGFR3* (2.5%; 31/1233) were the most frequent recurring kinases identified, but with different frequencies in ADC and SCC (Fig. 1b and Supplementary Table 1). *ALK* fusions were the most common among all histologic subtypes (Supplementary Table 1 and Supplementary Table 2). Compared to ADC, the frequencies of *ROS1* and *RET* were lower in SCC, and accounted for 8.5% and 2.9%, respectively. The prevalence of *FGFR3* was much higher in SCC than ADC (SCC vs. ADC: 28.6% vs. 1.5%, respectively).

We also summarized the number of kinase fusions carried by each patient (Fig. 1c). As expected, 94.7% of patients contained only one fusion, while 5.3% of patients harbored at least two fusions. The *ALK* gene rearrangement was most frequently observed in patients with either single fusion event or multiple fusions (59.6% and 80%, respectively). We also identified one patient with four kinase fusions that all belonged to *KIF5B-RET*, but with different fusion breakpoints.

We further analyzed the gender distribution in patients with different fusions (a sample size of more than 15 cases was required for each fusion) (Supplementary Fig. 1a). Most patients with *CCDC6-RET* and *FGFR3-TACC3* fusions were male, while the frequency of females was higher among patients with *VCL-ALK* and *SDC4-ROS1* fusions. Age was also statistically associated with fusion type. The ages of patients with *ROS1* fusions involving different partners varied significantly, as patients with *EZR-ROS1* were older than patients with *CD74*-, *SLC34A2*-, and *SDC4-ROS1* fusions (Supplementary Fig. 1b).

### Characterizing recurrent kinase fusions in lung cancer patients

**Fig. 2:**
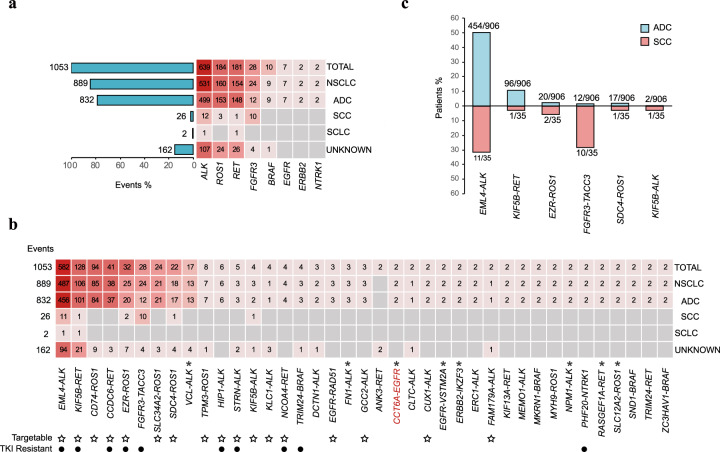
Recurrent kinase fusions in lung cancer. **a** Kinases involved in fusions in different lung cancer subtypes and the number of events in each lung cancer subtype is shown to the left of each bar. **b** Kinase fusions in different lung cancer subtypes and the number of events in each lung cancer subtype is shown on the left. The fusion in red is a novel kinase fusion that has not been previously reported in all cancer types. Fusions marked with * represent kinase fusions never reported in lung cancer. The ☆ showed the targetable kinase fusion and the • represent the fusion which was identified as resistance mechanism to EGFR TKI or fusion TKI. **c** Comparison of the prevalence of kinase fusions in ADC and SCC. The number of patients with each fusion and the total number of patients in ADC or SCC are shown on the top of each bar.

Recurrent kinase fusions have been of great interest and regarded as potential therapeutic targets for cancer treatment. Among 219 kinase fusion types, we observed 39 recurrent fusions involving 8 kinases, including *ALK*, *ROS1*, *RET*, *FGFR3*, *BRAF*, *EGFR*, *ERBB2*, and *NTRK1* (Fig. 2a, b). The most frequently occurring kinase was *ALK* (60.7%; 639/1053) and included *EML4-ALK* (91.1%; 582/639), *HIP1-ALK* (0.9%; 6/639), and *STRN-ALK* (0.8%; 5/639) fusions, and has been well documented in NSCLC^5–7^ (Fig. 2b). We also found several recurrent *ALK* fusions which have not been reported in lung cancer, but have been identified in other cancer types, such as *VCL-ALK*, *FN1-ALK*, and *NPM1-ALK*. Those data indicated that such rarely-reported driver fusions may also play crucial roles in the carcinogenesis of lung cancer. For the recurrent kinase fusions with low frequency (two occurrences) in our data, *CUX1-ALK*^8^ and *FAM179A-ALK*^9^ have been shown response to ALK TKIs. Meanwhile, it has been reported that genetic rearrangements can mediate drug resistance to TKI treatment^10^. *FGFR3-TACC3*^11,12^ is the resistance mechanism against EGFR TKIs in NSCLC patients with *EGFR* L858R or 19del mutation. *EML4-ALK*^13^ and *NCOA4-RET*^14^ are mechanisms of resistance to EGFR TKIs in NSCLC patients with *EGFR* T790M. Meanwhile, *KIF5B-RET*^15^, *CCDC6-RET*^16^, *EZR-ROS1*^17^, *PHF20-NTRK1*^18^, and *TRIM24-BRAF*^19^ have been reported to function as resistance mechanisms to *EGFR* 19del upon EGFR TKIs treatment in NSCLC patients. Besides, *HIP1-ALK*^20^ and *STRN-ALK*^21^ have been reported to mediate resistance to ALK TKIs in NSCLC patients with other *ALK* rearrangement. Further studies should be conducted to deeply understand the function of kinase fusions in cancer therapy.

Seven types of recurrent *ROS1* fusions were identified in our data, including the well described *CD74-ROS1*, *EZR-ROS1*, *SDC4-ROS1*, *SLC34A2-ROS1*, *TPM3-ROS1,* and *MYH9-ROS1*^22,23^, as well as a novel recurrent fusion, *SLC12A2-ROS1*, which was identified as a novel fusion in inflammatory myofibroblastic tumors (Fig. 2b). This was the first time that *SLC12A2-ROS1* was reported in lung cancer. For *RET* fusions, *KIF5B-RET* and *CCDC6-RET*, accompanied with other rarely-reported fusions, such as *NCOA4-RET*^24^, *ANK3-RET*, *KIF13A-RET*^25^, *RASGEF1A-RET*^26^, and *TRIM24-RET*^27^ were identified (Fig. 2b). Among which, *RASGEF1A-RET*^26^ was previously identified in breast cancer as a tumorigenic fusion sensitive to RET inhibitors, but not previously reported in lung cancer. Other recurrent fusions including *BRAF*, *EGFR*, *ERBB2*, and *NTRK1* were also observed. Among them, *EGFR-VSTM2A*, *CCT6A-EGFR*, and *ERBB2-IKZF3* have not previously been documented in lung cancer.

Importantly, only six recurrent fusions, including *EML4-ALK*, *KIF5B-RET*, *EZR-ROS1*, *FGFR3-TACC3*, *SDC4-ROS1*, and *KIF5B-ALK* were detected in ADC and SCC (Fig. 2c). Notably, *FGFR3-TACC3* was the only fusion involving *FGFR3* in this study and had a higher frequency in SCC patients (28.9%; 10/35) compared to ADC patients (1.3%; 12/906) (Fig. 2c).

### Breakpoint patterns of known kinase fusions

Although the kinases in our cohort all retained the integrity of the kinase domain, the breakpoints were variable when ligated with different partners. Such an observation may be caused by the structural and functional characteristics of each partner gene and may influence the tumorigenesis capacities of the fusions.

**Fig. 3:**
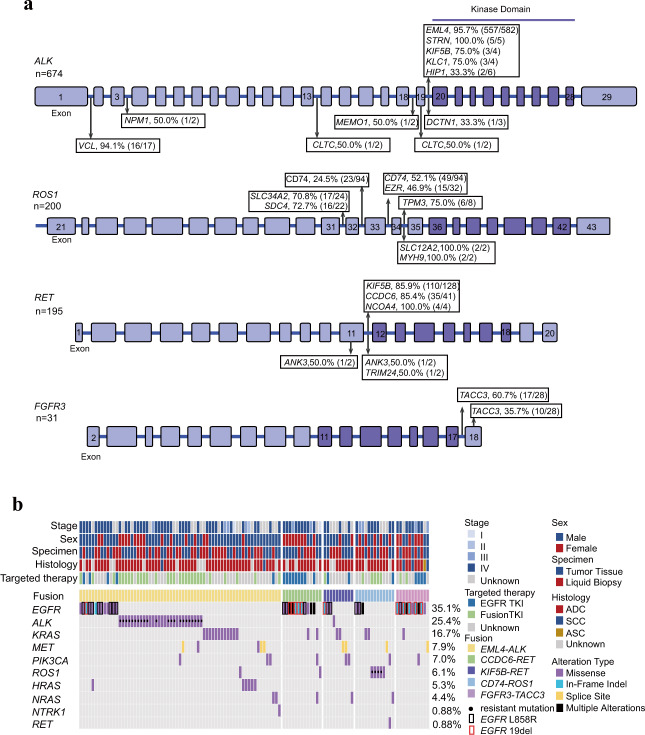
The breakpoint locations for kinase genes and druggable driver mutations co-occurring with kinase fusions. **a** The breakpoints for *ALK* with different partners; the breakpoints for *ROS1* with different partners; the breakpoints for *RET* with different partners; the breakpoints for *FGFR3* with different partners. The partners shown above the structure of each kinase belong to common partners. Some rare partners are shown under the kinase structure. The number of events involving each of the four kinases is shown to the left of each kinase structures. The percentage breakpoints with each partner, the number of events for each fusion in specific kinase breakpoints, and the total number of events for each fusion are shown. **b** The co-occurrence of kinase fusions and driver mutations is displayed in the hotspot plot. The incidences of driver gene mutations are shown to the left of the plot. Tissue/plasma samples were collected from 59 patients after developing resistance to EGFR TKI or fusion targeted treatment.

In *STRN-* (100.0%; 5/5), *EML4-* (95.7%; 557/582), *KIF5B-* (75.0%; 3/4), and *KLC1-ALK* (75.0%; 3/4) fusions, the breakpoints of *ALK* were almost exclusively in intron 19, which was consistent with previous studies^28–31^ (Fig. 3a). Interestingly, we identified a patient with a novel *ALK* breakpoint in intron 4 of a *KIF5B-ALK* fusion. Additionally, the *ALK* breakpoints in *VCL-ALK* fusions were primarily located in intron 1 (94.1%; 16/17), which was an uncommon breakpoint among *ALK* fusions and indicated a distinct rearrangement mechanism. Several novel breakpoints were also identified, including *CLTC-ALK* intron 13/exon 19 and *MEMO1-ALK* intron 18. The function of those fusion types should be further studied.

The most frequent fusion partner genes for *ROS1* were *CD74*, *EZR*, *SLC34A2*, *SDC4*, and *TPM3* (Supplementary Table 3). Although *CD74* was the most common partner in lung cancer, only *EZR-ROS1* and *SDC4-ROS1* fusions were found in SCC (Supplementary Table 3). The majority of breakpoints located in common positions of *ROS1* have been reported previously^32,33^ and we observed consistent results in our data. *CD74* (52.1%; 49/94) and *EZR* (46.9%; 15/32) were primarily ligated to intron 33 of *ROS1* (Fig. 3a). For most *SDC4-* (72.7%; 16/22) and *SLC34A2-ROS1* (70.8%; 17/24) fusions, the *ROS1* breakpoints were in intron 31, whereas *TPM3-ROS1* (75.0%; 6/8) fusions mainly occurred in intron 34 of *ROS1*. We also identified several rare events with *ROS1* breakpoints in intron 26 (*SLC34A2-ROS1*), intron 28 (*CD74-ROS1* and *SLC34A2-ROS1*), and exon 28 (*CD74-ROS1*). *ROS1* breakpoints involved in novel fusions with *SLC12A2* and *MYH9* were also in intron 34.

We also analyzed the most frequent fusion partner genes of *RET*, including *KIF5B*, *CCDC6*, and *NCOA4*, and found that they were primarily rearranged in intron 11 of *RET* at frequencies of 85.9% (110/128), 85.4% (35/41), and 100% (4/4), respectively (Fig. 3a). *TACC3* was the main fusion partner of *FGFR3* in both ADC (85.7%; 12/14) and SCC (100.0%; 10/10) (Supplementary Table 3), which frequently ligated to intron 17 (60.7%; 17/28) and exon 18 (35.7%; 10/28) of *FGFR3* (Fig. 3a and Supplementary Table 3).

### Analysis of mutations co-occurring with kinase fusions

The frequency of mutations co-occurring with *CCDC6-RET* (31.7%; 13/41) and *FGFR3-TACC3* (39.3%; 11/28) was higher than those co-occurring with *EML4-ALK* (11.5%; 67/582), *KIF5B-RET* (7.8%; 10/128), and *CD74-ROS1* (13.8%; 13/94) (Supplementary Fig. 2a). Strikingly, deep analysis of the concurrent mutations with *CCDC6-RET* and *FGFR3-TACC3* showed significant enrichment in *EGFR* ex19del-mutant patients (*p* < 0.01), respectively (Supplementary Fig. 2b and Fig. 3b) but not in L858R mutant patients. Indeed, most patients with *CCDC6-RET* and *FGFR3-TACC3* received prior *EGFR* TKI treatment; thus, suggesting that *CCDC6-RET* and *FGFR3-TACC3* may function as resistance mechanisms to *EGFR* TKIs in lung cancer, and may also highlight the differences in acquired resistance to TKIs through gene fusions between *EGFR* L858R and ex19del-mutant variants.

In the *EML4-ALK* group, *L858R* more frequently co-occurred than other *EGFR* mutations (Fig. 3b). Additionally, the frequency of *ALK* mutations was 44.3% (31/70), including *ALK* G1202R, G1269A, and L1196M, which are well-described resistance mechanisms to ALK TKIs. The *HRAS* mutation was only identified in the *EML4-ALK* group. *MET* exon 14 skipping (77.8%; 7/9) occurred significantly more frequently than other types of *MET* alterations that co-occurred with kinase fusions.

Interestingly, an ADC patient harboring an *EML4-ALK* fusion also had a *NTRK1 G595R* mutation, which is considered to be an acquired resistant mutation to *NTRK* inhibitors (larotrectinib or entrectinib)^34^. However, whether this alteration is associated with *ALK* TKI resistance remains to be determined. We also found a patient carrying *CD74-ROS1* and *RET V804M* alterations, however, the function of the concurrent *RET V804M* mutation requires further investigation.

### *RET* fusions in lung cancer and the response to RET inhibitors

**Fig. 4:**
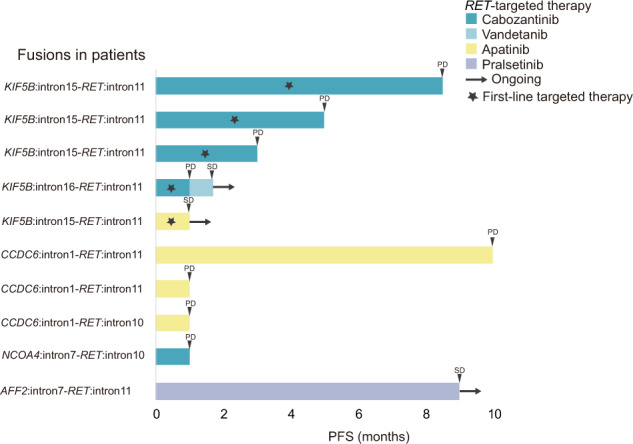
Progression Free Survival (PFS) of patients carrying *RET* fusions. Swimmer plot showing treatment drugs and durations (months). Patients with different fusions are indicated.

Since *RET* is a crucial therapeutic target in cancers, several multi-kinase inhibitors (MKIs) such as cabozantinib, vandetanib, alectinib, and apatinib have shown anti-RET activities. However, these MKIs have limited efficacy and patients with *RET* fusions showed poor responses with short progression free survival (PFS) times of generally less than 7.5 months^27^. In our cohort, targeted therapy information was available for 10 patients carrying different *RET* fusions (Fig. 4), among which nine patients were treated with MKIs and one was treated with the *RET*-selective inhibitor, pralsetinib. For the nine patients who received MKI therapy, five showed no response. Among the remaining four patients, three *KIF5B-RET*-positive cases received cabozantinib treatment and one achieved a PFS longer than 6 months. There were three *CCDC6-RET*-positive patients treated with apatinib in this study, but only one showed a response and achieved a PFS of 10 months. We also identified a patient with a novel *AFF2-RET* fusion who was treated with pralsetinib and maintained a response for 9 months. The clinical history of that *AFF2-RET* patient is described below.

### Novel fusions that demonstrated favorable clinical benefits of kinase inhibitors

**Fig. 5:**
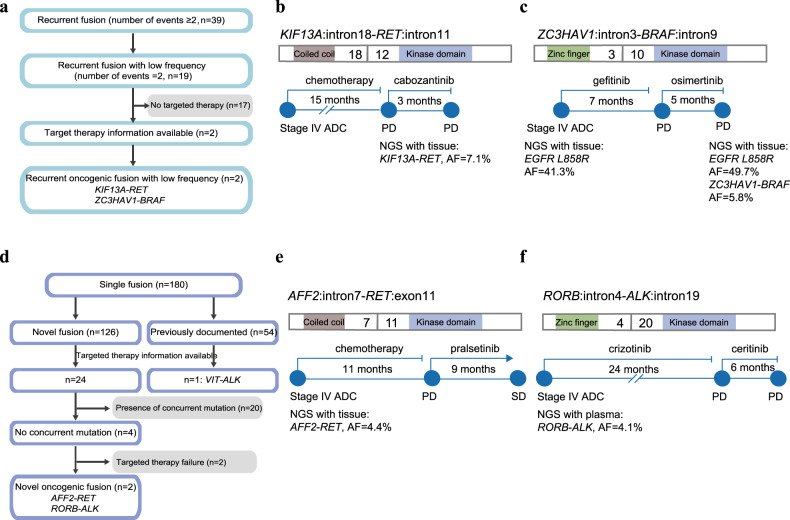
Potentially druggable fusions in lung cancer patients. **a** Pipeline of oncogenic fusions among all recurrent fusions. **b** The structure of KIF13A-RET and clinical information of the patient harboring that fusion. The domains are highlighted in different colors as: coil-coiled domain of KIF13A (brown) and RET tyrosine kinase domain (blue). **c** The structure of ZC3HAV1-BRAF and clinical information of the patient harboring that fusion. The domains are highlighted in different colors as: zinc finger domain of ZC3HAV1 (green) and BRAF tyrosine kinase domain (blue). **d** Pipeline of novel oncogenic fusions among single fusions. **e** The structure of AFF2-RET and clinical information of the patient harboring that fusion. The domains are highlighted in different colors as: coil-coiled domain of AFF2 (brown) and RET tyrosine kinase domain (blue). **f** The structure of RORB-ALK and clinical information of the patient harboring that fusion. The domains are highlighted in different colors as: zinc finger domain of RORB (green) and ALK tyrosine kinase domain (blue).

Besides the well-described recurrent kinase fusions, we also identified 19 recurrent fusion types with low frequency (two occurrences) in our cohort (Fig. 2). To search for functional fusions, we focused only on cases with targeted therapy information available, which included two fusions, *KIF13A-RET* and *ZC3HAV1-BRAF* (Fig. 5a). The patient with the *KIF13A-RET* fusion was a 64-year-old male with stage IV ADC. After 15 months of chemotherapy, the patient’s tumor biopsy was subjected to NGS and revealed the potential driver fusion, *KIF13A*: exon18*-RET*: exon12. Subsequently, the patient was treated with cabozantinib, but failed to exhibit a response (Fig. 5b). The patient harboring the *ZC3HAV1-BRAF* fusion was a 56-year-old female with stage IV ADC. Her tissue and plasma biopsies were subjected NGS which identified the *EGFR L858R* mutation at mutation allele frequency (MAF) of 41.3% and 3.0%, respectively. The patient was administered gefitinib and achieved a PFS of 7 months. After the disease progressed, the patient was administered osimertinib treatment, but only responded for 5 months. The tumor progressed and an additional metastatic site was identified, and thus, the patient’s plasma and metastatic tissue biopsies were subjected to a second NGS test. That test identified a *ZC3HAV1*: exon3*-BRAF*: exon10 mutation at a MAF of 3.1% and 5.8%, respectively, thus, indicating the resistance function of the *ZC3HAV1-BRAF* fusion (Fig. 5c).

We also found 180 types of single kinase fusions, including 126 fusions that had not previously been reported. To scan for functional cases from novel kinase fusions, we evaluated the therapeutic history of each patient and focused only on fusions without concurrent driver mutations that benefited from fusion TKI treatments (Fig. 5d). We identified two novel fusion types, including *AFF2-RET* (*AFF2*: intron7-*RET*: exon11) and *RORB-ROS1* (*RORB*: intron4-*ALK*: intron19). The patient harboring the *AFF2-RET* fusion was a male with stage IV ADC. His plasma biopsy was subjected to NGS, and the novel *AFF2-RET* fusion was identified at a MAF of 4.1%. After chemotherapy for 11 months, the patient received pralsetinib treatment and achieved a stable disease, which was maintained for 9 months. Additional investigations of the AFF domain identified a coil-coiled domain, which is capable of mediating dimerization (Fig. 5e). The patient carrying the *RORB-ALK* fusion was a 64-year-old male with stage IV ADC. His tumor biopsy was subjected to NGS, which revealed the potential driver fusion. Subsequently, the patient was treated with crizotinib and achieved a response for 24 months. After disease progression, the patient was treated with ceritinib and responded for 6 months. Structural analysis of RORB revealed a zinc finger domain that may lead to dimerization and activation of downstream ALK targets (Fig. 5f). These two novel kinase fusions both exhibited remarkable sensitivity to *RET* and *ALK* TKIs, respectively, which suggested that they were able to function as potentially druggable fusions.

## Discussion

In this study, we used DNA-seq data from 17,442 Chinese lung cancer patients to identify potential driver fusions, and analyze the clinical and genomic features of patients with different kinase fusion types. Overall, 6.66% (1162/17,442) of patients contained kinase fusions (recurrent fusions account for 5.2%). Of the 219 fusion types observed, 39 were recurrent while 180 were observed in single cases. The most frequent kinases identified in patients were *ALK*, *RET*, *ROS1*, *EGFR*, and *FGFR3*.

An interesting observation was the identification of recurrent fusions that had not previously been reported in lung cancer, but were found in other cancer types, such as the *VCL-ALK* fusion in epithelioid fibrous histiocytoma^35^ and renal cell carcinoma^36^, the *FN1-ALK* fusion in ovarian cancer^37^ and gastrointestinal leiomyomas^38^, the *NPM1-ALK* fusion in anaplastic large cell lymphoma^39^, the *VSTM2A-EGFR* fusion in glioblastomas^40^, and the *ERBB2-IKZF3* fusion in breast cancer^41^. Those results indicated that driver fusions in other cancers may also play crucial roles in lung cancer carcinogenesis. We also identified a novel recurrent fusion, *CCT6A*-*EGFR*, but the function of which remains to be determined.

Many driver mutations often co-occurred and played critical roles with kinase fusions during cancer progression. Thus, detailing the relationships between fusions and mutations may lead to the development of efficient treatment strategies. Therefore, we analyzed treatment-relevant driver alterations that co-occurred with the most frequent kinase fusions.

Further investigation of the breakpoints involved in different kinase fusions revealed preferences for the ligation sites of partners. Most of the fusions occurred in intron 11 and intron 17 of *RET* and *FGFR3*, respectively, but the breakpoints of *ALK* and *ROS1* varied between different partners. Studies showed that translocations were not random, as the breakpoints in the fusion genes had both sequence and structure preferences^42^. It was reported that *ALK* fusion variants may affect clinical outcomes upon TKI treatment^43^.

By integrating mutation, copy number variation, and fusion data, we evaluated the genomic alterations co-occurring with each fusion, and focused mainly on alterations related to drug administration. Patients with *CCDC6-RET* and *FGFR3-TACC3* had a higher prevalence of *EGFR* ex19del mutations (*p* < 0.01), which indicated that they may function as resistance mechanisms for *EGFR* TKIs. It has been reported that *CCDC6-RET* can mediate osimertinib resistance in NSCLC, and the combination of osimertinib and *RET* inhibitors leads to a rapid response in those patients^44^. In our cohort, nine patients with *EGFR* alterations were treated with *EGFR* TKIs, among which seven were treated with osimertinib. However, two patients with *CCDC6-RET* were only treated with gefitinib and ectinib, respectively, which indicated that the *CCDC6-RET* fusion functioned as a resistance alteration against first and third generation TKIs. The therapeutic approach to targeting those fusions should be further studied. *FGFR3-TACC3* could confer resistance to EGFR TKIs and often had co-existing EGFR activating mutations in NSCLC^11^, which is consistent with our findings. We also revealed that the frequency of patients with *MET*ex 14 skipping was higher than that of other *MET* alterations co-occurring with kinase fusions. However, the underlying mechanisms leading to such observations should be studied in detail.

Since only 6% of cancer patients can benefit from the existing drugs targeting fusions, the identification of novel druggable fusions is crucial for expanding the therapeutic options for cancer patients. In this study, we focused only on fusions with intact kinase domains, without concurrent driver mutations, and studied the clinical history of each patient. We identified two novel fusions, including *AFF2-RET* and *RORB-ALK* with demonstrated responses to TKI treatment. We analyzed the domains of the two fusions and found that they both harbored domains that mediate protein dimerization and activate downstream signaling pathways. We also observed a patient with a novel *AFF3-ALK* fusion, without the dimerization domain (data not shown). As expected, that patient showed no response when treated with crizotinib.

This study has two main limitations. The first one is that the therapeutic information were incomplete or missing, which is also one of the weaknesses of real-world studies^45^. Although we used a system with error alert function to abstract medical record, errors might have occurred during information collection. The other limitation is that we did not validate the novel or concurrent fusions via other diagnostic approaches including reverse transcription polymerase chain reaction, or RNA sequencing which due to the insufficiency of tissue amount. A recent study^46^ showed that two non-canonical ALK fusions identified with DNA sequencing in a patient generated canonical EML4-ALK transcripts during mRNA maturation which was revealed via RNA sequencing, indicating a difference of fusion detected at DNA and RNA level. Therefore, other diagnostic approaches should be used for further validation of fusions observed with DNA sequencing.

In conclusion, we provided a comprehensive overview of the landscape of kinase fusions in a large Chinese lung cancer population, including lung ADC and SCC patients, and characterized the patterns of breakpoint locations and co-occurring mutations in patients with known kinase fusions. Furthermore, we reported novel kinase fusions and highlighted patients with recurrent kinase fusions with low frequency (two occurrences) that demonstrated favorable clinical outcomes on TKIs. Collectively, these findings not only advance our understanding of the spectrum of fusions involving kinases in lung cancer, but also have immediate implications for disease diagnosis and treatment.

## Methods

### Patients and samples

Using capture-based targeted NGS, this study retrospectively reviewed 17,442 Chinese NSCLC patients who underwent genetic testing at hospitals across China between June 2016 and July 2019, including The Second Affiliated Hospital of Zhejiang University School of Medicine, Fujian Medical University Cancer Hospital and Zhangzhou Zhengxing Hospital. Demographic and clinical data, including age, gender, histology type, pathological stage, metastasis sites, treatment regimens, and duration of TKI treatment were abstracted from the medical records provided by physicians. Genomic profiling was performed on formalin-fixed paraffin-embedded (FFPE) tumor and liquid biopsy specimens. All samples were sequenced in a Clinical Laboratory Improvement Amendments (CLIA)- and College of American Pathologists (CAP)-certified genomic testing facility (Nanjing Geneseeq Technology Inc., Nanjing, China). This study was approved by the ethical committee of each participating hospital and all patients provided written informed consent to participate.

### Library preparation and sequencing

For targeted NGS, DNA extraction and sequencing libraries were prepared according to protocols described previously^47^. Briefly, genomic DNA was extracted from FFPE tumor and liquid biopsies using a QIAamp DNA FFPE Tissue Kit and a QIAamp Circulating Nucleic Acid kit (Qiagen), respectively. Library preparations were performed using the KAPA Hyper Prep Kit (KAPA Biosystems). Target enrichment was performed using the xGen lockdown probes targeting 425 cancer-related genes (Geneseeq Prime). The target-enriched libraries were quantified by qPCR using the KAPA Library Quantification kit (KAPA Biosystems) and sequenced on HiSeq NGS platforms (Illumina) to generate 2 × 150-bp reads following the manufacturer’s instructions.

### Fusions detection and kinase fusion analysis

We used Delly fusion calling tool^48^ to identify the number of chimeric reads (sequencing paired ends mapped to different genes) and split reads (spanning a fusion breakpoint) from the targeted DNA-seq data. Fusions were filtered by removing fusions with (1) breakpoints involving intergenic loci; and (2) fusions with split reads <3 or paired reads <5. Then, kinase fusions matched to the Kinase Database (including 538 kinase genes; http://kinase.com/web/current/) were evaluated with the conservation of full catalytic kinase domains. All fusions were manually confirmed using the Integrative Genomics Viewer (IGV) and only kinase fusions retaining an intact kinase domain were included for analysis in this study. For patients with multiple tests, same fusion was counted only once regardless of whether it was detected in multiple samples or different sample sources. Samples’ therapeutic timelines, including treatment-naïve and post-TKI treatment, were considered unless this information was lacking.

### Mutation calling

Somatic alterations concurrent with kinase fusions were analyzed as previously described^49^. In brief, we used Trimmomatic for FASTQ file quality control (below 15 or N bases were removed). Burrows–Wheeler Aligner (BWA v0.7.12) was used for mapping reads to the reference Human Genome (hg19). Local realignment around the indels and base quality score recalibration was performed with the Genome Analysis Toolkit (GATK 3.4.0). VarScan 2 was used for somatic mutations calling, with at least 0.2% MAF and with at least three supporting-reads. The Oncology Knowledge Base (oncoKB) was used to identify oncogenic alterations^50^. Information on targeted therapy-relevant hotspot alterations were also abstracted from the oncogenic alterations^51^.

### Data analysis and statistics

The chi-squared test, Mann–Whitney *U* test, and Kruskal–Wallis *H* test were used for analyses of gender and age. A *p*-value < 0.05 was considered significant for all tests, unless otherwise indicated. Statistical analyses were performed in R (v.3.3.2) and Statistical Product and Service Solutions.

### Reporting summary

Further information on research design is available in the Nature Research Reporting Summary linked to this article.

## Supplementary information

Supplementary Information

Reporting Summary

## Data Availability

The data presented in the study are deposited in the Genome Sequence Archive for Human (GSA-Human) repository, accession number HRA001045.

## Code availability

Chimeric reads were called with Delly tool (v0.7.8; available at https://github.com/dellytools/delly). Reads were mapped to the reference Human Genome with Burrows–Wheeler Aligner (BWA) (v0.7.12; available at https://github.com/lh3/bwa/). Local realignment around the indels and base quality score recalibration was applied with the Genome Analysis Toolkit (GATK) (v3.4.0; available at https://software.broadinstitute.org/gatk/). The code that supports the findings of this study are available from the corresponding authors on reasonable request.
