# Supplementary material for: Genomic characterization and outcome evaluation of kinome fusions in lung cancer revealed novel druggable fusions
Source: NPJ Precis Oncol. 2021 Sep 10;5:81. doi: 10.1038/s41698-021-00221-z (PMC8433182; doi:10.1038/s41698-021-00221-z)
Supplement: Supplementary file 1 — Supplementary Information [file 41698_2021_221_MOESM1_ESM.pdf]

Genomic characterization and outcome evaluation of kinome fusions in lung cancer revealed novel druggable fusions

Supplementary Information

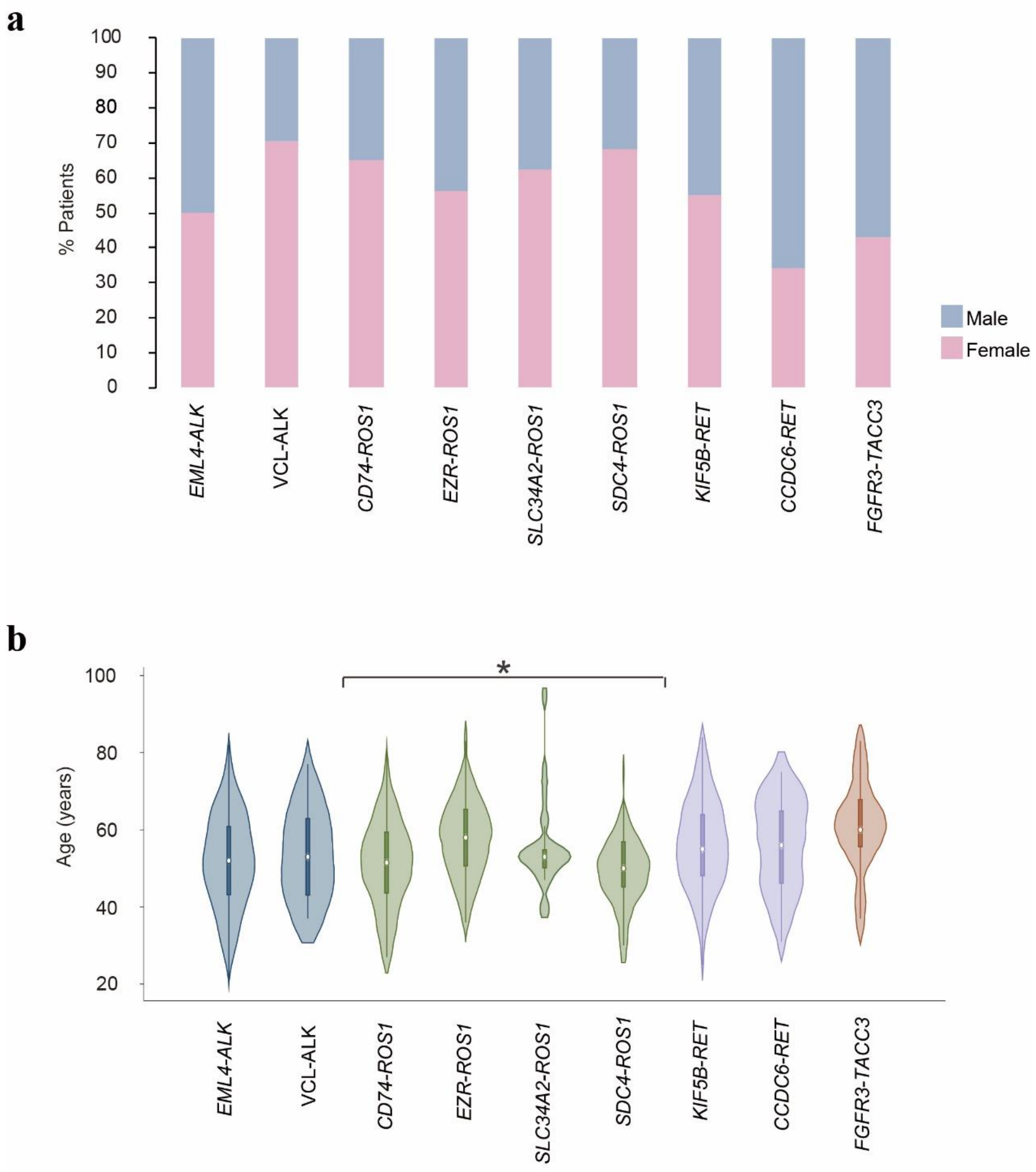

Supplementary Figure 1. The distribution of gender (a) and age (b) of patients carrying different kinase fusions. *, P-value (Kruskal-Wallis H Test) < 0.05.

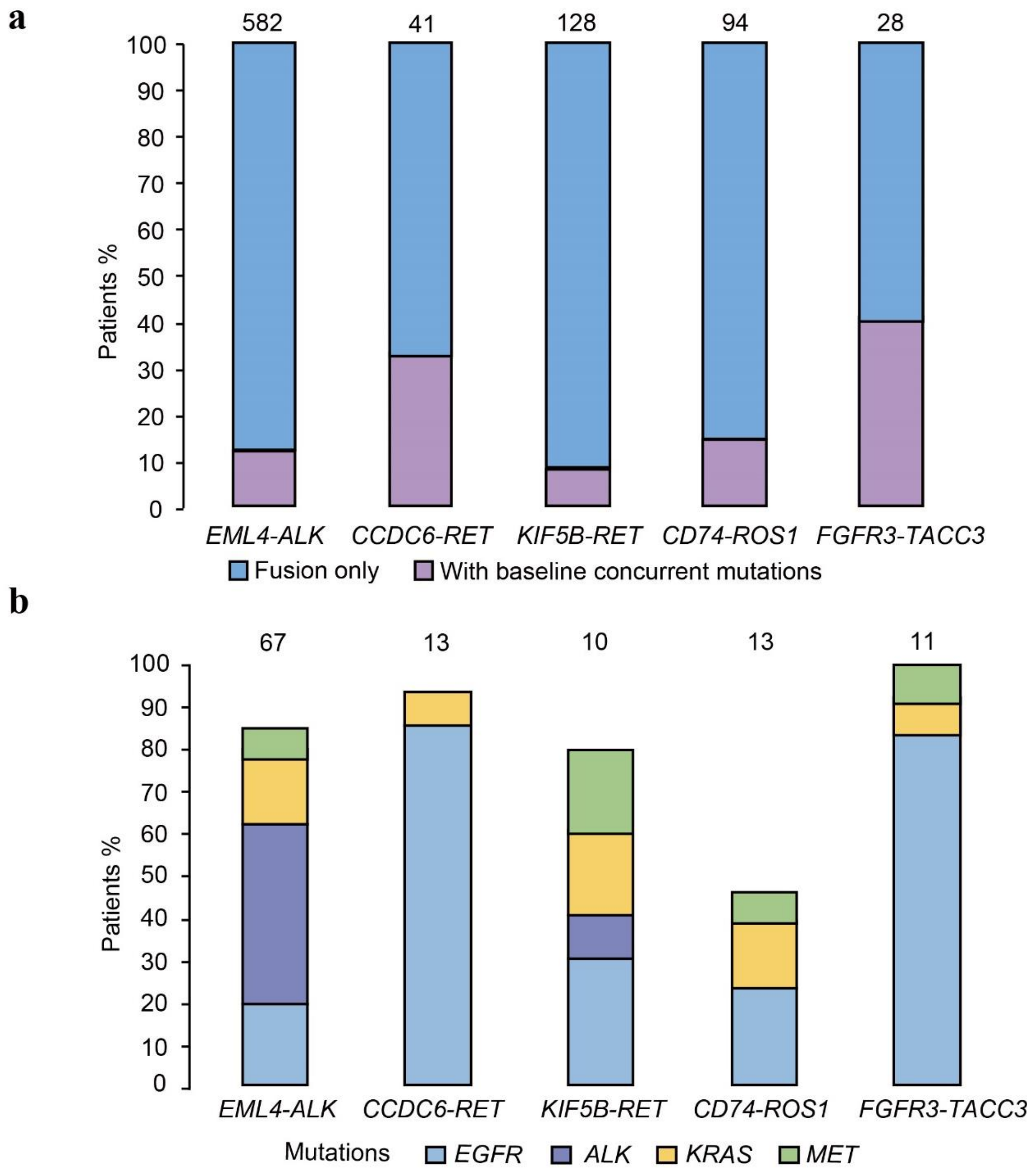

Supplementary Figure 2. The baseline concurrent mutations and common mutations co-occurring with kinase fusions. a The baseline concurrent mutations for patients carrying different kinase fusions. The number of patients with each kinase fusion is shown on the top of each bar. **b** The common mutations co-occurring with kinase fusions. The number of patients with each kinase fusion is shown on the top of each bar.

Supplementary Table 1. The proportion of all kinase genes in all events

	All	NSCLC					SCLC	Unknown
		ADC	SCC	ASC	LCC	Unknown		
The number of events	1233	964	35	10	4	22	5	193
Kinase Genes, n (%)								
ALK	674 (54.8)	526 (54.5)	13 (37.0)	4 (40.0)	2 (50.0)	14 (63.6)	2 (40.0)	113 (58.6)
ROS1	200 (16.2)	167 (17.3)	3 (8.5)	1 (10.0)	1 (25.0)	2 (9.1)	0	26 (13.5)
RET	195 (15.8)	159 (16.5)	1 (2.9)	1 (10.0)	0	4 (18.2)	2 (40.0)	28 (14.5)
FGFR3	31 (2.5)	14 (1.5)	10 (28.6)	2 (20.0)	0	0	0	5 (2.6)
BRAF	21 (1.7)	18 (1.9)	0	0	0	0	0	3 (1.6)
EGFR	20 (1.6)	17 (1.8)	1 (2.9)	0	0	0	0	2 (1.0)
ERBB2	17 (1.4)	15 (1.6)	1 (2.9)	0	0	0	1 (20.0)	0
NTRK1	7 (0.6)	6 (0.6)	0	0	0	0	0	1 (0.5)
FGFR1	6 (0.5)	4 (0.4)	1 (2.9)	0	0	0	0	1 (0.5)
CHEK2	5 (0.4)	4 (0.4)	1 (2.9)	0	0	0	0	0
FGFR2	4 (0.3)	2 (0.2)	0	1 (10.0)	0	0	0	1 (0.5)
KIT	3 (0.2)	1 (0.1)	0	0	0	0	0	2 (1.0)
PDGFRB	3 (0.2)	1 (0.1)	0	0	0	0	0	2 (1.0)
RAF1	3 (0.2)	2 (0.2)	1 (2.9)	0	0	0	0	0
Others	44 (3.6)	28 (2.9)	3 (8.5)	1 (10.0)	1 (25.0)	2 (9.1)	0	9 (4.7)

Abbreviations: NSCLC, non-small-cell lung cancer; SCLC, small cell lung cancer; ADC, adenocarcinoma; SCC, squamous cell carcinoma; ASC, adenosquamous carcinoma; LCC, large cell carcinoma.

Supplementary Table 2. The proportion of all kinase genes in 17442 patients

	All	NSCLC					SCLC	Unknown
		ADC	SCC	ASC	LCC	Unknown		
Patients	17442	10932	1353	162	63	217	549	4166
Kinase Genes, n (%)								
ALK	641 (3.675)	500 (4.574)	13 (0.961)	4 (2.469)	2 (3.175)	14 (6.452)	2 (0.364)	106 (2.544)
ROS1	196 (1.124)	163 (1.491)	3 (0.222)	1 (0.617)	1 (1.587)	2 (0.922)	0	26 (0.624)
RET	185 (1.061)	151 (1.381)	1 (0.074)	1 (0.617)	0	4 (1.843)	2 (0.364)	26 (0.624)
FGFR3	31 (0.178)	14 (0.128)	10 (0.739)	2 (1.235)	0	0	0	5 (0.120)
BRAF	21(0.120)	18 (0.165)	0	0	0	0	0	3 (0.072)
EGFR	18 (0.103)	15 (0.137)	1 (0.074)	0	0	0	0	2 (0.048)
ERBB2	16 (0.092)	14 (0.128)	1 (0.074)	0	0	0	1 (0.182)	0
NTRK1	7 (0.040)	6 (0.055)	0	0	0	0	0	1 (0.024)
FGFR1	6 (0.034)	4 (0.037)	1 (0.074)	0	0	0	0	1 (0.024)
CHEK2	5 (0.029)	4 (0.037)	1 (0.074)	0	0	0	0	0
FGFR2	4 (0.023)	2 (0.018)	0	1* (0.617)	0	0	0	1 (0.024)
KIT	2 (0.011)	1 (0.009)	0	0	0	0	0	1 (0.024)
PDGFRB	2 (0.011)	1 (0.009)	0	0	0	0	0	1 (0.024)
RAF1	3 (0.017)	2 (0.018)	1 (0.074)	0	0	0	0	0
Others	26 (0.149)	11 (0.101)	3 (0.222)	1* (0.617)	1 (1.587)	2 (0.922)	0	8 (0.192)

*: The same patient.

Abbreviations: NSCLC, non-small-cell lung cancer; SCLC, small cell lung cancer; ADC, adenocarcinoma; SCC, squamous cell carcinoma; ASC, adenosquamous carcinoma; LCC, large cell carcinoma.

Supplementary Table 3. Common partners of the main kinase genes in lung cancer patients

	Total	ADC	SCC
Events of *ALK* fusions	674	526	13
Common partner genes of *ALK*, events (%)	618 (91.7)	481 (91.4)	12 (92.3)
EML4	582 (86.4)	456 (86.7)	11 (84.6)
VCL	17 (2.5)	13 (2.5)	0
HIP1	6 (0.9)	6 (1.1)	0
STRN	5 (0.7)	3 (0.6)	0
KIF5B	4 (0.6)	2 (0.4)	1 (7.7)
KLC1	4 (0.6)	1 (0.2)	0
Events of *ROS1* fusions	200	167	3
Common partner of *ROS1*, events (%)	180 (90.0)	149 (89.2)	3 (100.0)
CD74	94 (47.0)	84 (50.3)	0
EZR	32 (16.0)	20 (12.0)	2 (66.7)
SLC34A2	24 (12.0)	21 (12.6)	0
SDC4	22 (11.0)	17 (10.2)	1 (33.3)
TPM3	8 (4.0)	7 (4.2)	0
Events of *RET* fusions	195	159	1
Common partner of *RET*, events (%)	173 (88.7)	142 (89.3)	1 (100.0)
KIF5B	128 (65.6)	101 (63.5)	1 (100.0)
CCDC6	41 (21.0)	37 (23.3)	0
NCOA4	4 (2.1)	4 (2.5)	0
Events of *FGFR3* fusions	31	14	10
Common partner of *FGFR3*, events (%)	28 (90.3)	12 (85.7)	10 (100.0)
TACC3	28 (90.3)	12 (85.7)	10 (100.0)

Abbreviations: ADC, adenocarcinoma; SCC, squamous cell carcinoma.
