# Supplementary material for: Genomic characterization and outcome evaluation of kinome fusions in lung cancer revealed novel druggable fusions
Source: NPJ Precis Oncol. 2021 Sep 10;5:81. doi: 10.1038/s41698-021-00221-z (PMC8433182; doi:10.1038/s41698-021-00221-z)
Supplement: Supplementary file 2 — Reporting Summary [file 41698_2021_221_MOESM2_ESM.pdf]

nature research

Corresponding author(s): Nong Lin

Last updated by author(s): Mar 3, 2021

Reporting Summary

Nature Research wishes to improve the reproducibility of the work that we publish. This form provides structure for consistency and transparency in reporting. For further information on Nature Research policies, see our Editorial Policies and the Editorial Policy Checklist.

Statistics

For all statistical analyses, confirm that the following items are present in the figure legend, table legend, main text, or Methods section.

n/a	Confirmed	
☒	☐	The exact sample size (*n*) for each experimental group/condition, given as a discrete number and unit of measurement
☐	☒	A statement on whether measurements were taken from distinct samples or whether the same sample was measured repeatedly
☐	☒	The statistical test(s) used AND whether they are one- or two-sided<br>*Only common tests should be described solely by name; describe more complex techniques in the Methods section.*
☒	☐	A description of all covariates tested
☒	☐	A description of any assumptions or corrections, such as tests of normality and adjustment for multiple comparisons
☐	☒	A full description of the statistical parameters including central tendency (e.g. means) or other basic estimates (e.g. regression coefficient) AND variation (e.g. standard deviation) or associated estimates of uncertainty (e.g. confidence intervals)
☒	☐	For null hypothesis testing, the test statistic (e.g. *F*, *t*, *r*) with confidence intervals, effect sizes, degrees of freedom and *P* value noted<br>*Give P values as exact values whenever suitable.*
☒	☐	For Bayesian analysis, information on the choice of priors and Markov chain Monte Carlo settings
☒	☐	For hierarchical and complex designs, identification of the appropriate level for tests and full reporting of outcomes
☒	☐	Estimates of effect sizes (e.g. Cohen's *d*, Pearson's *r*), indicating how they were calculated

Our web collection on statistics for biologists contains articles on many of the points above.

Software and code

Policy information about availability of computer code

Data collection	Chimeric reads were called with Delly tool (v0.7.8; available at https://github.com/dellytools/delly). Reads were mapped to the reference Human Genome with Burrows-Wheeler Aligner (BWA) (v0.7.12; available at https://github.com/lh3/bwa/). Local realignment around the indels and base quality score recalibration was applied with the Genome Analysis Toolkit (GATK) (v3.4.0; available at https://software.broadinstitute.org/gatk/). The code that supports the findings of this study are available from the corresponding authors on reasonable request.
Data analysis	Chimeric reads were called with Delly tool (v0.7.8; available at https://github.com/dellytools/delly). Reads were mapped to the reference Human Genome with Burrows-Wheeler Aligner (BWA) (v0.7.12; available at https://github.com/lh3/bwa/). Local realignment around the indels and base quality score recalibration was applied with the Genome Analysis Toolkit (GATK) (v3.4.0; available at https://software.broadinstitute.org/gatk/). The code that supports the findings of this study are available from the corresponding authors on reasonable request.

For manuscripts utilizing custom algorithms or software that are central to the research but not yet described in published literature, software must be made available to editors and reviewers. We strongly encourage code deposition in a community repository (e.g. GitHub). See the Nature Research guidelines for submitting code & software for further information.

Data

Policy information about availability of data

All manuscripts must include a data availability statement. This statement should provide the following information, where applicable:

- Accession codes, unique identifiers, or web links for publicly available datasets
- A list of figures that have associated raw data
- A description of any restrictions on data availability

The datasets generated and/or analysed during the current study are available from the corresponding author on reasonable request.

Field-specific reporting

Please select the one below that is the best fit for your research. If you are not sure, read the appropriate sections before making your selection.

☒ Life sciences ☐ Behavioural & social sciences ☐ Ecological, evolutionary & environmental sciences

For a reference copy of the document with all sections, see nature.com/documents/nr-reporting-summary-flat.pdf

Life sciences study design

All studies must disclose on these points even when the disclosure is negative.

Sample size	The sample size was determined by samples available.
Data exclusions	No data were excluded from the analyses.
Replication	Not applicable.
Randomization	Not applicable.
Blinding	Not applicable.

Reporting for specific materials, systems and methods

We require information from authors about some types of materials, experimental systems and methods used in many studies. Here, indicate whether each material, system or method listed is relevant to your study. If you are not sure if a list item applies to your research, read the appropriate section before selecting a response.

Materials & experimental systems

n/a	Involved in the study	
☒	☐	Antibodies
☒	☐	Eukaryotic cell lines
☒	☐	Palaeontology and archaeology
☒	☐	Animals and other organisms
☐	☒	Human research participants
☒	☐	Clinical data
☒	☐	Dual use research of concern

Methods

n/a	Involved in the study	
☒	☐	ChIP-seq
☒	☐	Flow cytometry
☒	☐	MRI-based neuroimaging

Human research participants

Policy information about studies involving human research participants

Population characteristics	1162 Chinese lung cancer patients with kinase fusions were analyzed (median age:53) . Clinical information of these patients is shown in Table 1.
Recruitment	Patients were recruited through the participating institutions: The Second Affiliated Hospital of Zhejiang University School of Medicine, Fujian Medical University Cancer Hospital and Zhangzhou Zhengxing Hospital.
Ethics oversight	This study was approved by the ethical committee of each participating hospital.

Note that full information on the approval of the study protocol must also be provided in the manuscript.
